# Gene Regulation Network of Prognostic Biomarker YAP1 in Human Cancers: An Integrated Bioinformatics Study

**DOI:** 10.3389/pore.2021.1609768

**Published:** 2021-06-11

**Authors:** Baojin Wu, Xinjie Tang, Honglin Ke, Qiong Zhou, Zhaoping Zhou, Shao Tang, Ronghu Ke

**Affiliations:** ^1^ Department of Plastic Surgery, Huashan Hospital, Fudan University, Shanghai, China; ^2^ Department of Emergency, Huashan Hospital Affiliated to Fudan University, Shanghai, China; ^3^ Department of Statistics, Florida State University, Tallahassee, FL, United States

**Keywords:** cancer, prognosis, YAP1, bioinformatic, mitochondrial

## Abstract

**Background:** Yes-associated protein 1 (YAP1) is the main downstream effector of the Hippo signaling pathway, which is involved in tumorigenesis. This study aimed to comprehensively understand the prognostic performances of YAP1 expression and its potential mechanism in pan-cancers by mining databases.

**Methods:** The YAP1 expression was evaluated by the Oncomine database and GEPIA tool. The clinical significance of YAP1 expression was analyzed by the UALCAN, GEPIA, and DriverDBv3 database. Then, the co-expressed genes with YAP1 were screened by the LinkedOmics, and annotated by the Metascape and DAVID database. Additionally, by the MitoMiner 4.0 v tool, the YAP1 co-expressed genes were screened to obtain the YAP1-associated mitochondrial genes that were further enriched by DAVID and analyzed by MCODE for the hub genes.

**Results:** YAP1 was differentially expressed in human cancers. Higher YAP1 expression was significantly associated with poorer overall survival and disease-free survival in adrenocortical carcinoma (ACC), brain Lower Grade Glioma (LGG), and pancreatic adenocarcinoma (PAAD). The LinkedOmics analysis revealed 923 co-expressed genes with YAP1 in adrenocortical carcinoma, LGG and PAAD. The 923 genes mainly participated in mitochondrial functions including mitochondrial gene expression and mitochondrial respiratory chain complex I assembly. Of the 923 genes, 112 mitochondrial genes were identified by MitoMiner 4.0 v and significantly enriched in oxidative phosphorylation. The MCODE analysis identified three hub genes including CHCHD1, IDH3G and NDUFAF5.

**Conclusion:** Our findings showed that the YAP1 overexpression could be a biomarker for poor prognosis in ACC, LGG and PAAD. Specifically, the YAP1 co-expression genes were mainly involved in the regulation of mitochondrial function especially in oxidative phosphorylation. Thus, our findings provided evidence of the carcinogenesis of YAP1 in human cancers and new insights into the mechanisms underlying the role of YAP1 in mitochondrial dysregulation.

## Introduction

Cancer is among the most common causes of morbidity and mortality worldwide and its incidence and mortality are rapidly growing, particularly in less developed countries. Previous observation showed that over 1.5 million individuals were diagnosed with malignant tumors and 0.6 million individuals died of cancer each year in the United States [1]. Based on the mortality, cancer has become one of the most threatening diseases for human health. Despite the rapid improvement of diagnosis and anti-cancer therapies in recent years, the 5-years survival rate of patients remains unsatisfactory for various types of tumors [2]. Thus, it is important that we further investigate the mechanism of carcinogenesis and explore the potential biomarkers for early diagnosis, accurate prognosis, and targets for therapy in cancers.

Yes-associated protein 1 (YAP1) is a well-characterized transcription co-activator and main downstream effector of the Hippo signaling pathway, which is involved in tissue repair and regeneration, as well as tumorigenesis. YAP was observed to promote cell growth and restrain apoptosis, acting as an oncogene promoter [3]. Subsequently, YAP1 was reported to be associated with tumor progression and patients’ prognosis in various kinds of cancers including bladder cancer, breast cancer, gastric cancer, head and neck cancers, colorectal cancer, and pancreatic ductal adenocarcinoma (PAAD) [4–9]. In contrast, YAP1 was shown to be involved in cell apoptosis and coordinate cell proliferation and cell death during development [10]. In promyelocytic leukemia, YAP1 was further shown to induces apoptosis in response to DNA damage, indicating that YAP might play a role in tumor suppression [11]. Thus, the role of YAP1 in cancer development remains controversial, and further study of the clinical significance of YAP1 expression is needed.

Recently, the availability of multiple omics data provides a new opportunity to investigate the pathogenic mechanism and to improve the treatment of cancer. In particular, some latest studies focus on the multiplatform-based analysis of the YAP1 prognostic valve in specific cancers, such as Zhou et al [9] focused on pancreatic cancer, Guichet [12] et al focused on glioma, Giraud et al [13]focused on gastric cancer. These studies indicated important opportunities for targeted and combination therapies of cancer. However, most of these studies only focused on one specific tumor. Currently, relatively little is known of the landscape of YAP1 clinical significance in pan-cancers and the identification of featured molecular mechanisms.

In the present study, we comprehensively analyzed YAP1 expression and its association with prognostic values in pan-cancers *via* multiplatform such as the Oncomine, GEPIA, UALCAN, and DriverDBv3. Our study revealed the significant prognostic potential of YAP1 in adrenocortical carcinoma (ACC), brain Lower Grade Glioma (LGG), and pancreatic adenocarcinoma (PAAD). Additionally, we identified the YAP1-associated genes and constructed a YAP1-associated PPI network that is specific to mitochondria in ACC, LGG and PAAD. Our results showed mitochondrial dysfunction (oxidative phosphorylation) was significantly involved in the three types of tumors and three hub genes (CHCHD1, IDH3G and NDUFAF5) were identified. Thus, our findings provided evidence of the prognosis values of YAP1 in human cancers and new insights into the mechanism underlying the function of YAP1 in mitochondrial dysfunction.

## Materials and Method

### Analysis of the YAP1 Expression in Human Cancers

To analyze the YAP expression in different types of human cancers, the Oncomine and GEPIA online databases were used for the visualization of gene expression. The Oncomine database is the largest oncogene chip database and data mining tool that includes 715 gene expression datasets from 86,733 caners and normal samples [14]. The expression differences of the YAP1 mRNA between tumors and normal tissues in distinct types of cancer were determined within the Oncomine database. The thresholds of deregulated YAP1 expression were used by the default cutoff in the Oncomine, which including *p*-values less than 1E-4 and Fold change (FC) greater than 1.5. GEPIA (http://gepia.cancer-pku.cn/) is a web server for cancer and normal gene expression profiling and interactive analyses based on 9,736 tumors and 8,587 normal samples from the TCGA (The Cancer Genome Atlas) and the GTEx (Genotype-Tissue Expression) databases [15]. In the study, YAP1 high or low expression was identified by the default cutoff in the GEPIA, which including *p*-values less than 0.01 and |Log2FC| greater than 1.

### Prognostic Potential of YAP1 Expression in Cancers

Following the analysis of the YAP expression in human cancers, the prognostic potential of YAP1 expression was evaluated by using the GEPIA, UALCAN, and DriverDBv3 online databases [16]. As mentioned above, GEPIA database provided key interactive and customizable functions including patient survival analysis. The correlations between YAP1 expression and survival, including overall survival (OS) and disease-free survival (DFS), were also analyzed by GEPIA. The HR and *p* or Cox *p*-values from a log-rank test were included in the plot. The UALCAN is an interactive web portal to perform in-depth analyses of gene expression and clinical data from The Cancer Genome Atlas (TCGA) database (http://ualcan.path.uab.edu). In this study, we used the UALCAN database to explore the associations between the YAP1 expression levels and patients’ survival in distinct types of cancers. The DriverDBv3 is a cancer omics database that incorporates RNA expression, miRNA expression, methylation, copy number variation, and somatic mutation [17]. Here, the DriverDBv3 was used to examine the prognostic potential of YAP1 transcriptional levels in different human cancers. Survival-relevant with log-rank *p*-value <0.05 was considered significant.

### Analysis of the YAP1 Co-Expressed Genes by the LinkedOmics Database

The LinkedOmics database (http://www.linkedomics. org/login. php) is a web-based platform for analyzing 32 TCGA cancer-associated multi-dimensional datasets. The LinkFinder module was used to investigate genes that were differentially expressed in correlation with YAP1 in ACC, LGG, and PAAD. In the module, the RNA-seq datasets (HiSeq RNA) from TCGA samples were selected and then analyzed by Spearman’s correlation test. The YAP1 co-expressed genes were listed by Spearman’s correlation coefficient and represented in volcano plots. The positive or negative correlation genes were screened by the *p*-value (*p* < 0.01) [18]. The correlation genes in each tumor were intersected to obtain the common YAP1-correlated genes across the three types of tumors (ACC, LGG, and PAAD).

### Functional Enrichment Analysis *Via* Metascape and DAVID Database

Following the screening of the common YAP1-correlated genes, the Metascape tool was used for further gene annotation analysis. The Metascape was applied to perform GO and KEGG pathway analysis. GO term analysis includes biological processes (BP), cellular components (CC), and molecular function (MF) [16]. To validate the annotation for the YAP1 co-expressed genes, we further used the DAVID (the database for Annotation, Visualization, and Integrated Discovery) online bioinformatics database. The DAVID is an analysis tool of biological data to integrate and provide information for biological function. This tool was also used to provide GO enrichment and KEGG pathway analysis in this study. The *p*-value <0.05 was considered as statistical significance.

### Mitochondrial Gene Network Analysis

The YAP1 co-expressed genes were filtered for mitochondrial identity by using the MitoMiner 4.0 v tool. The mitochondrial genes were defined by the integrated mitochondrial protein index as “KNOWN” mitochondrial. These “KNOWN” mitochondrial genes were firstly annotated by using the DAVID as mentioned above. Subsequently, these “KNOWN” mitochondrial genes were imported into STRING database to construct the mitochondrial gene network. The k-means network was built using an interaction score set at a confidence of 0.7 using the whole human genome as a background statistical Ref. [19]. In our study, the mitochondrial network was further visualized by Cytoscape software (version 3.4.0). Then, the Cytoscape plugin MCODE was used to screen the hub gene. The parameters of MCODE were set as follows: find clusters = in whole network, degree cutoff = 2, cluster finding = haircut, node score cutoff = 0.2, k-core = 2 and max depth = 100. The parameters of cytoHubba were set as follows: Hubba nodes = top 10 nodes ranked by degree, display options = check the first-stage nodes, display the shortest path, and display the expanded subnetwork [20].

### Statistics Analysis

The YAP1 expression levels and its prognostics values in various tumors were analyzed *via* public databases. The default statistical methods were chosen when there is no special description.

## Results

### The Expression Levels of YAP1 in Different Types of Human Cancers

**FIGURE 1:**
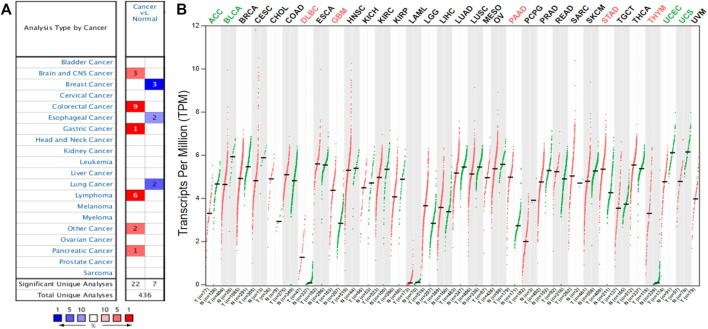
The YAP1 expression levels in different human cancers. The differential expression of YAP1 across all tumor samples and paired normal tissues in the Oncomine **(A)**. The color is determined by the gene percentile for the analyses within the cell. The comparison of YAP1 expression in different tumor tissues and normal tissues based on the GEPIA database **(B)**. The expression data were scaled by log2 (TPM+1).

To detect the differential expression of YAP1 in human cancers, the Oncomine database was used to obtain the YAP1 gene expression profile across tumor samples and paired normal tissues. This database included a total of 436 unique analyses for YAP1. YAP1 was upregulated in cancers based on 22 significant unique analyses and YAP1 was downregulated in seven unique analyses. YAP1 was overexpressed in colorectal cancer, lymphoma, brain and CNS cancer, gastric and pancreatic cancer. But lower YAP1 expression was observed in breast cancer, lung cancer, and esophageal cancer (Figure 1A). This result revealed that YAP1 was overexpressed in most cancers. To further validate the differential expression of YAP1 in different types of tumors, the GEPIA platform was applied to analyze the data of RNA sequencing from TCGA and GTEx datasets. As shown in Figure 1B, YAP1 was upregulated in DLBC (lymphoid neoplasm diffuse large B-cell lymphoma), GBM (glioblastoma multiforme), PAAD (pancreatic adenocarcinoma), STAD (stomach adenocarcinoma) and THYM (thymoma). However, the level of YAP1 expression was lower in ACC (adrenocortical carcinoma), BLCA (bladder urothelial carcinoma), UCEC (uterine corpus endometrial carcinoma) and UCS (uterine carcinosarcoma). These results also revealed YAP1 was upregulated in the majority of tumors including PAAD and lymphoma.

### Prognostic Potential of YAP1 in Different Cancers

Following the differential expression of YAP1 in different types of tumors, we firstly employed the GEPIA tool to analyze the prognostic performance of YAP1 in various cancers. According to the median mRNA levels of YAP1, Kaplan-Meier survival analysis was performed in pan-cancers. As for overall survival (OS), high levels of YAP1 could be considered an unfavorable biomarker for LGG patients (*n* = 514, Log-rank *p* = 0.0069, HR = 1.7; Supplementary Figure S1A), PAAD patients (*n* = 178, Log-rank *p* = 0.0053, HR = 1.8; Supplementary Figure S1B) and ACC patients (*n* = 76, Log-rank *p* = 0.0064, HR = 36; Supplementary Figure S1C). However, high levels of YAP1 expression were observed to be a favorable indicator for ESCA (Esophageal carcinoma) patients (*n* = 182, Log-rank *p* = 0.012, HR = 0.56; Supplementary Figure S1D). As for disease-free survival (DFS), high levels of YAP1 expression were associated with poorer prognosis in BLCA (*n* = 402, Log-rank *p* = 0.036, HR = 1.4; Supplementary Figure S1E) and ACC (*n* = 76, Log-rank *p* = 0.00018, HR = 3.7; Supplementary Figure S1F). In brief, except for ESCA, high YAP1 expression was associated with a poor prognosis in various kinds of tumors including ACC, PAAD, LGG, and BLCA.

**FIGURE 2:**
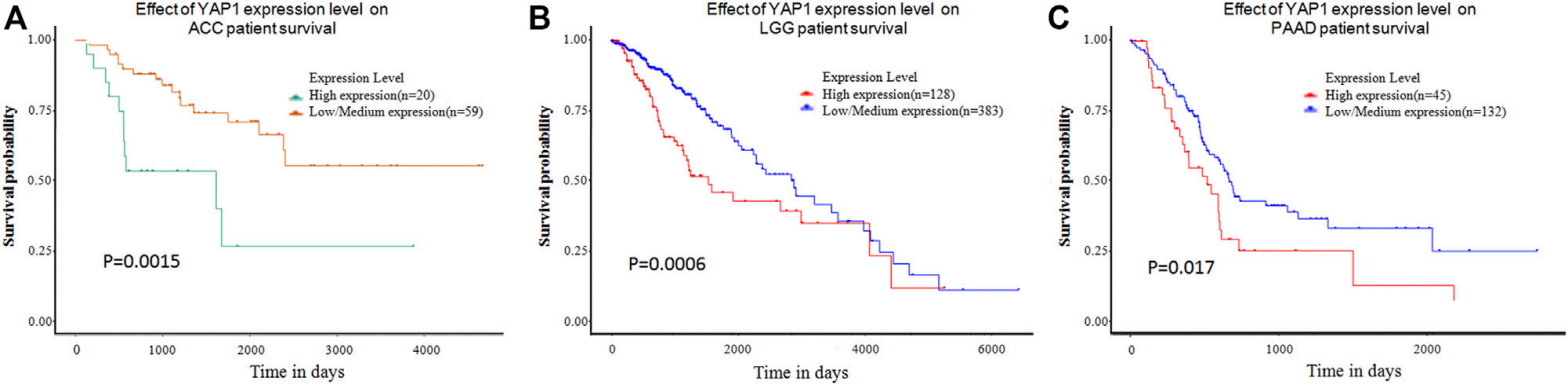
The effect of YAP1 expression level on tumor Patient survival from the UALCAN. YAP1 overexpression was correlated with poor survival in ACC **(A)**, LGG **(B)**, and PAAD **(C)**. ACC: adrenocortical carcinoma; LGG: brain Lower Grade Glioma; PAAD: pancreatic adenocarcinoma.

In addition to the GEPIA, UALCAN database was further used to evaluate the clinical significance of YAP1 expression in pan-cancers. In agreement with results from GEPIA, the UALCAN database also demonstrated high levels of YAP1 were shown to be an unfavorable marker for ACC patients (*n* = 79, *p* = 0.0015, Figure 2A), LGG patients (*n* = 511, *p* = 0.00063, Figure 2B) and PAAD patients (*n* = 177, *p* = 0.0015, Figure 2C).

**FIGURE 3:**
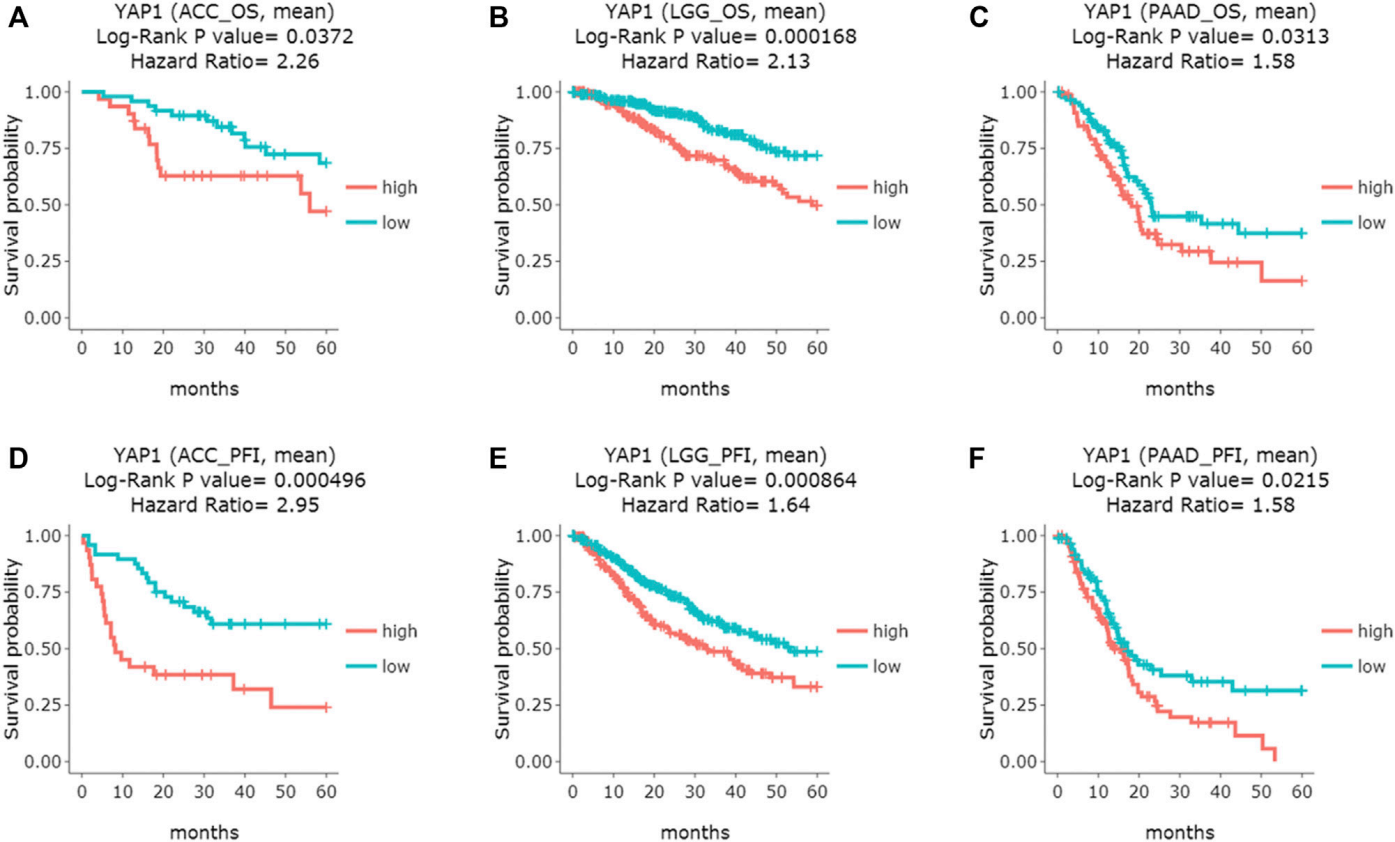
The prognostic curves of the YAP1 gene from DriverDBv3 database. YAP1 overexpression was correlated with poor overall survival (OS) in ACC **(A)**, LGG **(B)**, and PAAD **(C)**; Moreover, High YAP1 expression predicted poor progress-free interval (PFI) in ACC **(D)**, LGG **(E)** and PAAD **(F)**. ACC: adrenocortical carcinoma; LGG: brain Lower Grade Glioma; PAAD: pancreatic adenocarcinoma.

Additionally, the DriverDBv3 tool was also applied to determine the association between YAP1 expression and patients’ survival rates in pan-cancers. Consistent with the results from the above two platforms, DriverDBv3 tool showed higher level of YAP1 expression was correlated with shorter overall survival in ACC (Log-rank *p* = 0.0372, HR = 2.26; Figure 3A), LGG (Log-rank *p* = 0.000168, HR = 2.13; Figure 3B) and PAAD (Log-rank *p* = 0.0313, HR = 1.58; Figure 3C). Notably, high YAP1 expression was observed to be linked with shorter PFI in ACC (Log-rank *p* = 0.000496, HR = 2.95; Figure 3D), LGG (Log-rank *p* = 0.000864, HR = 1.64; Figure 3E) and PAAD (Log-rank *p* = 0.0215, HR = 1.58; Figure 3F). Altogether, these results suggested that YAP1 could be a potential prognostic biomarker in some specific types of cancers and YAP1 over expression was significantly associated with poor prognosis in ACC, LGG, and PAAD.

### YAP1 Co-Expression Networks in Specific Tumor

**FIGURE 4:**
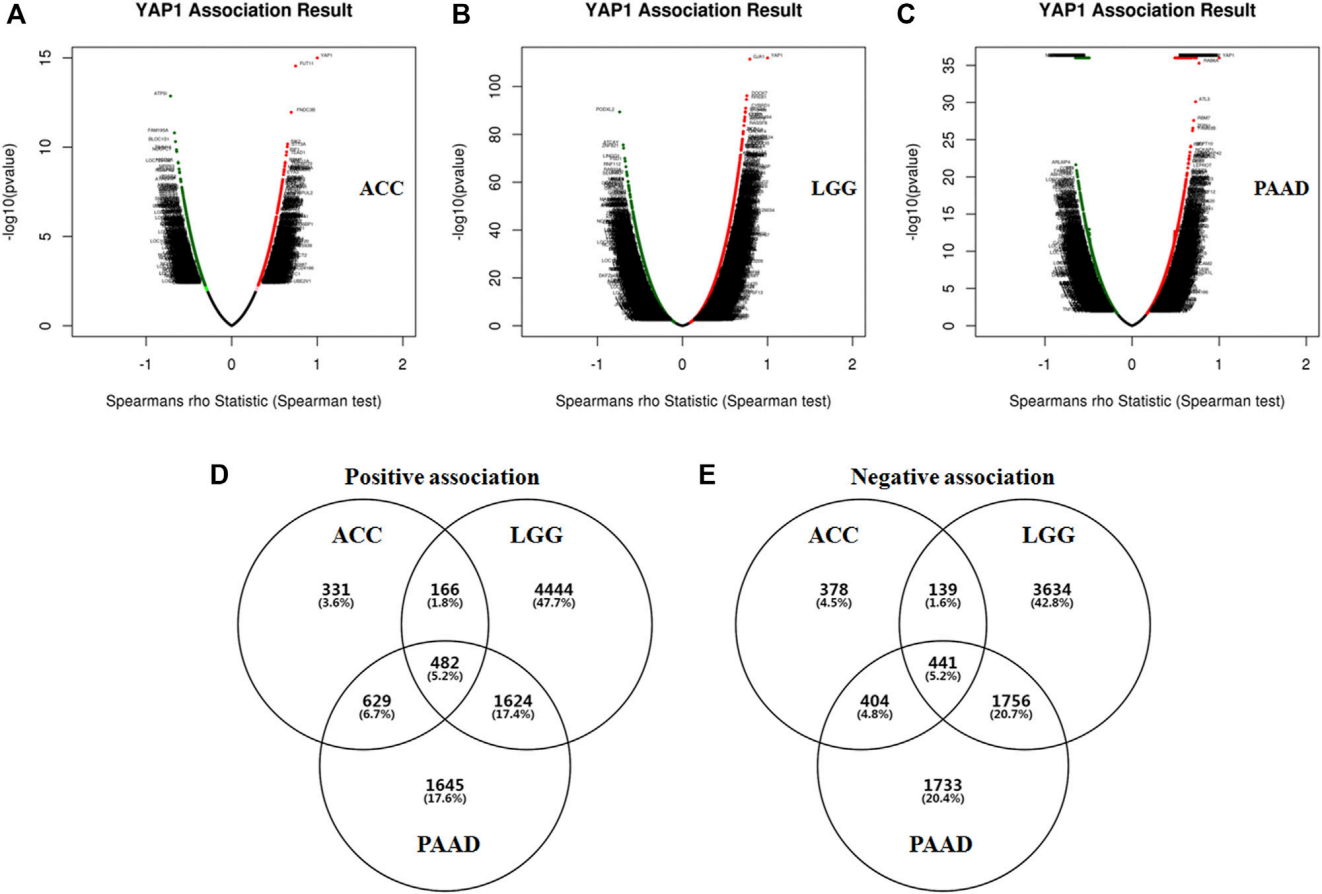
The co-expression genes of YAP1 in specific cancers (LinkedOmics). The YAP1 significantly correlated genes were analyzed by Pearson test in ACC **(A)**, LGG **(B)**, and PAAD **(C)**. Green and red dots represented the negative and positive correlations with YAP1, respectively. The common YAP co-expressed genes were intersected. The YAP1 positively **(D)** or negatively **(E)** correlated genes were identified in ACC, LGG, and PAAD.

Given the significant prognostic value of YAP1 in ACC, LGG and PAAD, the co-expressed genes with YAP1 in the three types of cancers were analyzed by using LinkedOmics to explore the potential mechanism. Firstly, the co-expressed genes in each tumor were identified by Spearman’s correlation test (*p* < 0.01). In ACC, 2,507 genes (dark red dots) were shown to have significant positive correlations with YAP1, whereas 2,216 genes (dark green dots) were shown to have significant negative correlations with YAP1 (Figure 4A). In LGG, 7,279 genes (red dots) were shown to have significant positive correlations with YAP1, whereas 6,553 genes (green dots) were shown significant negative correlations (Figure 4B). In PAAD, 3,558 genes were shown to have significant positive correlations with YAP1, whereas 1,891 genes were shown to have significant negative correlations (Figure 4C). Next, the above correlation genes with YAP1 in each tumor were intersected to obtain the common correlation genes across the three types of cancers. As shown in the chart (Figures 4D,E), a total of 482 genes were positively associated with YAP1, and 441 genes were negatively associated with YAP1 across the three types of cancers (Figures 4C,D). These common positive or negative correlation genes with YAP1 were detailed in Supplementary Tables S1, S2, respectively. The common correlation genes were used for further enrichment analysis.

### Functional Enrichment for the YAP1-Associated Genes

**FIGURE 5:**
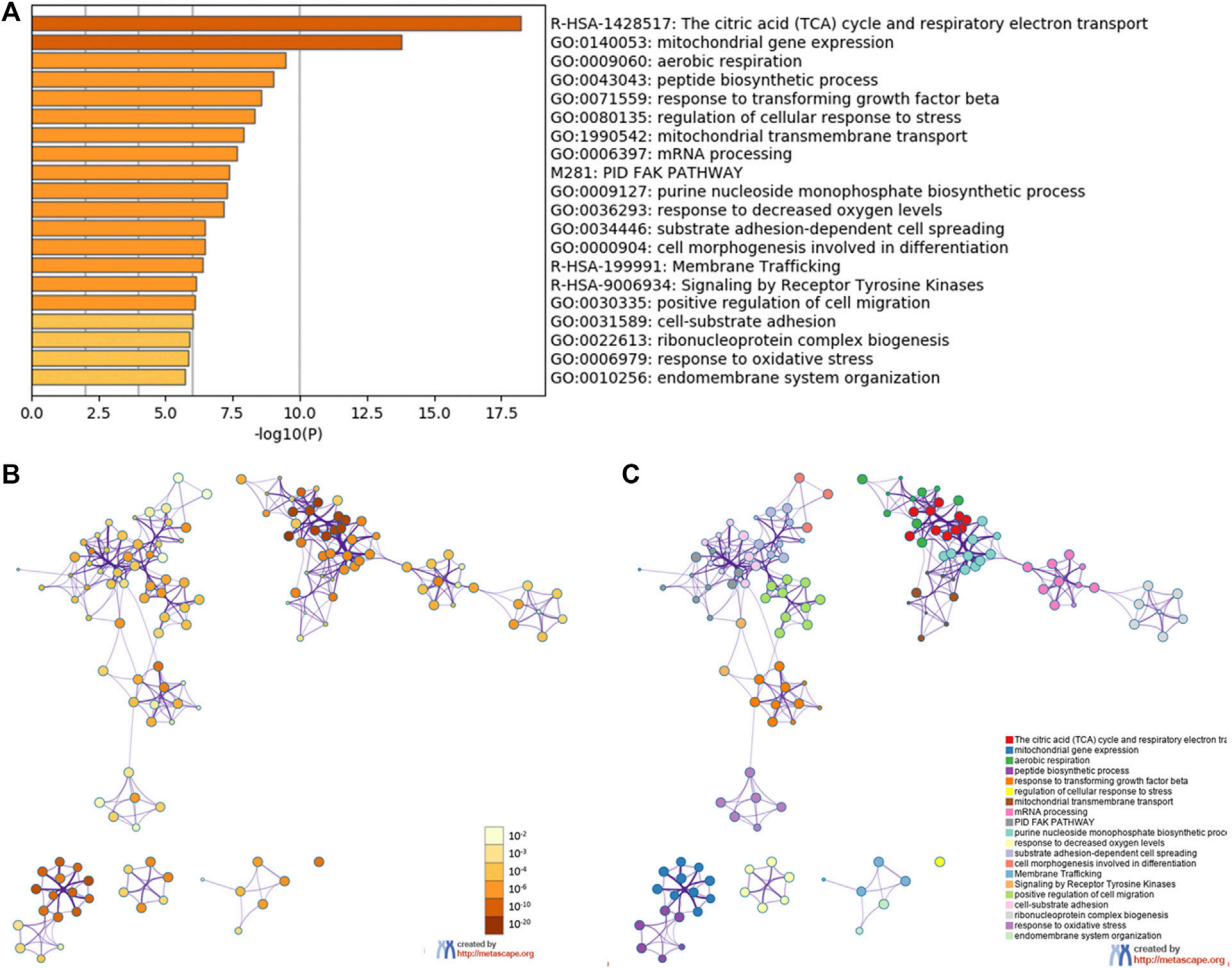
The enrichment analysis of YAP1 coexpression genes in Metascape database. The YAP1 coexpression genes were enriched by GO analysis including biological process, molecular function, and cellular component. The 20 most enrichment terms were shown **(A)**. Orange denoted the enrichment terms colored by *p* values **(B)**. An interactive network of the top 20 enrichment terms colored by cluster ID **(C)**. Different colors represented various enrichment pathways of the YAP1 coexpression genes.

To predict the functions of the co-expression genes with YAP1, we performed analyses of GO and KEGG pathways in the Metascape platform. As shown in Figure 5A, the YAP1co-expression genes were significantly involved in mitochondrial gene expression, aerobic respiration, peptide biosynthetic process, response to transforming growth factor-beta, regulation of cellular response to stress, mitochondrial transmembrane transport, and mRNA processing. As the signal pathway, these genes were significantly enriched in citric acid (TCA) cycle and respiratory electron transport, PID FAK pathway, and signaling by receptor tyrosine kinases. To visualize the pathways, Cytoscape was further used. Each node represented an enriched term and is colored by the cluster-ID. The enriched terms included mitochondrial gene expression, aerobic respiration, peptide biosynthetic process, response to transforming growth factor-beta, regulation of cellular response to stress, mitochondrial transmembrane transport, mRNA processing, and signaling pathways (TCA cycle and respiratory electron transport, PID FAK pathway, and signaling by receptor tyrosine kinases, Figures 5B,C).

**FIGURE 6:**
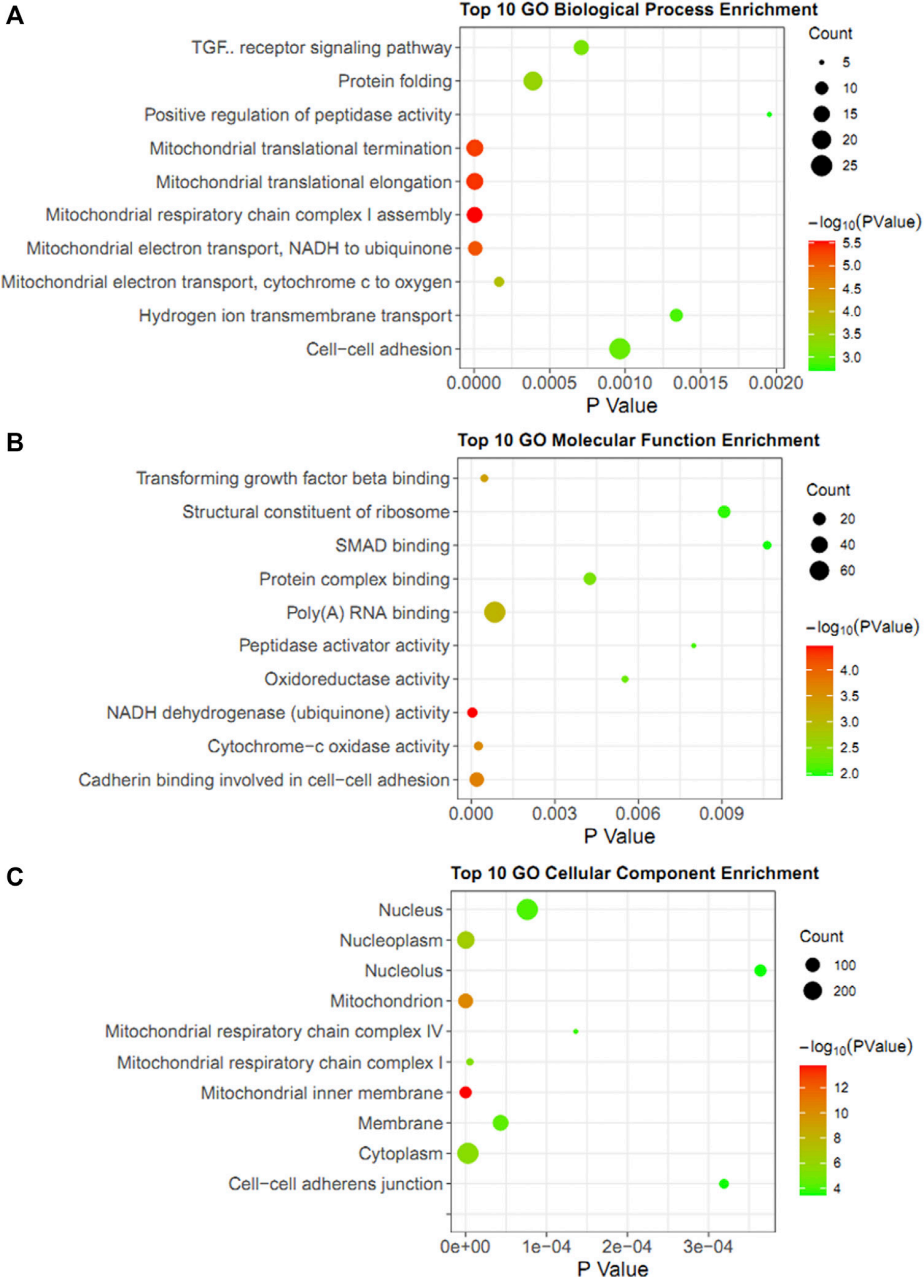
GO enrichment analysis of YAP1 coexpression genes in DAVID database. The bubble diagrams visualized the results of GO analysis for biological process **(A)**, molecular function **(B)**, and cellular components **(C)**. The size of the dot denoted the number of genes. The degrees of color represented *p*-value.

In addition to the Metascape tool, the DAVID database was also used to annotate the co-expression genes with YAP1. As for biological process enrichment, the YAP1 co-expressed genes were significantly involved in mitochondrial respiratory chain complex I assembly, mitochondrial translational elongation, mitochondrial translational termination, mitochondrial electron transport, NADH to ubiquinone, and mitochondrial electron transport, cytochrome c to oxygen (Figure 6A). As for molecular function enrichment, these genes primarily participated in NADH dehydrogenase (ubiquinone) activity, cadherin binding involved in cell-cell adhesion, cytochrome-c oxidase activity, and transforming growth factor-beta binding (Figure 6B). As for cellular component annotation, the YAP1 co-expressed genes were enriched in the mitochondrial inner membrane, mitochondrion, nucleoplasm, and mitochondrial respiratory chain complex I (Figure 6C). Altogether, the enrichment analysis from the two platforms indicated the YAP1 co-expressed genes were significantly associated with mitochondrial functions.

### Mitochondrial Gene Network Analysis of the YAP1-Associated Genes

**FIGURE 7:**
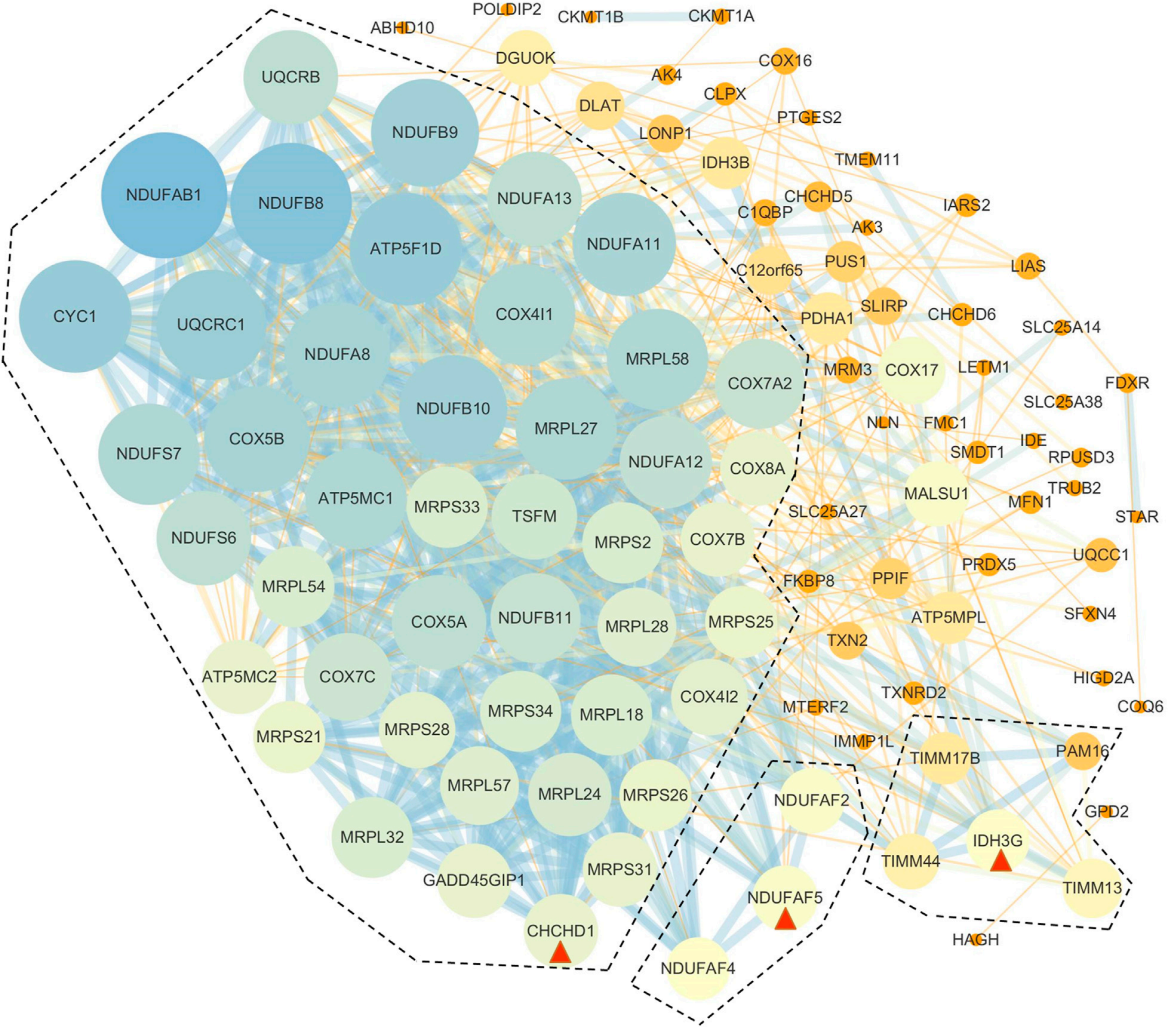
PPI networks of YAP1-associated mitochondrial genes. The PPI network of the genes was constructed by STRING and analyzed by MCODE. Three hub modules were identified and hub genes in each module were is circled red.

Based on the results of GO enrichment, the YAP1 co-expressed genes were focused on the mitochondrial networks. Among the YAP1 co-expressed genes, 112 mitochondrial genes were identified by using the MitoMiner 4.0 (Supplementary Table S3). By using String, our result showed the network of the mitochondrial genes consisted of 112 nodes and 783 edges (Figure 7). To construct the crucial sub-network, the plugin MCODE tool was used to calculate the most significant clusters in the sub-network [21]. As shown in Figure 7 and Supplementary Table S4, three clusters were identified in the network. Among the three clusters, cluster1 contained 43 clustered genes and its seed gene, CHCHD1. The cluster2 included four clustered genes and its seed gene, IDH3G. In contrast, cluster3 was composed of just three genes, including NDUFAF2, NDUFAF4 and NDUFAF5. To annotate the 112 mitochondrial genes, we performed analyses of the GO and KEGG pathways within the DAVID database. As shown in Table 1, the 112 mitochondrial genes were significantly involved in mitochondrion organization, cellular respiration, generation of precursor metabolites and energy, electron transport chain, mitochondrial gene expression, purine nucleoside triphosphate metabolic process, and mitochondrial translation. Subsequently, the significant KEGG pathway analysis showed the 112 mitochondrial genes were mainly enriched in oxidative phosphorylation (Table 2). The most significantly enriched pathway (the oxidative phosphorylation) was further visualized by the KOBAS online analysis database (Supplementary Figure S2).

**TABLE 1:** GO biological process enrichment for 112 mitochondrial genes.

Term	ID	Input gene count	Background gene count	False discovery rate	Matching proteins in your network (labels)
Mitochondrion organization	GO: 0007005	35	424	7.69E-27	ATP5F1D, TIMM13, PPIF, NDUFS7, COX17, NDUFB10, TIMM44, NDUFS6, NDUFB11, NDUFB9, IMMP1L, FMC1, CHCHD6, NDUFAF2, NDUFB8, PAM16, LETM1, TMEM11, NDUFA12, LONP1, NDUFAF4, NDUFA8, UQCC1, NDUFAF, COX16, TP5MC1, ATP5MC2, TIMM17B, NDUFA11, MFN1, NDUFA13, UQCRB, SLIRP, NDUFAB1, POLDIP2
Cellular respiration	GO: 0045333	25	153	3.25E-25	UQCRC1, IDH3G, NDUFS7, COX5B, NDUFB10, NDUFS6, NDUFB9, DLAT, NDUFAF2, NDUFB8, GPD2, CYC1, COX5A, COX8A, CHCHD5, NDUFA12, NDUFA8, COX4I2, PDHA1, IDH3B, COX7C, UQCRB, COX4I1, NDUFAB1, SLC25A14
Generation of precursor metabolites and energy	GO: 0006091	32	400	2.78E-24	UQCRC1, ATP5F1D, IDH3G, NDUFS7, COX5B, COX17, NDUFB10, NDUFS6, NDUFB9, DLAT, NDUFAF2, NDUFB8, GPD2, CYC1, COX5A, COX8A, CHCHD5, NDUFA12, PTGES2, COX7A2, NDUFA8, COX4I2, PDHA1, IDH3B, COX7C, TXNRD2, FDXR, COX7B, UQCRB, COX4I1, NDUFAB1, SLC25A14
Electron transport chain	GO: 0022900	24	169	4.35E-23	UQCRC1, NDUFS7, COX5B, NDUFB10, NDUFS6, NDUFB9, NDUFAF2, NDUFB8, GPD2, CYC1, COX5A, COX8A, NDUFA12, PTGES2, COX7A2, NDUFA8, COX4I2, IDH3B, TXNRD2, COX7B, COX7C, UQCRB, COX4I1, NDUFAB1
Mitochondrial gene expression	GO:0140,053	22	137	3.47E-22	MRPS34, MRPL28, MRPL32, MRPL27, C12orf65, MRPS25, MRPS28, TSFM, MRPL58, MRPL57, MRPS31, MRPL54, MRPL24, MRPL18, MRPS2, CHCHD1, PUS1, MRPS26, MRPS33, MTERF2, GADD45GIP1, MRPS21
Purine nucleoside triphosphate metabolic process	GO: 0009144	25	228	7.61E-22	UQCRC1, ATP5F1D, NDUFS7, COX5B, DGUOK, NDUFB10, NDUFS6, AK3, NDUFB9, NDUFB8, CLPX, COX8A, CYC1, COX5A, NDUFA12, NDUFA8, COX4I2, ATP5MC1, ATP5MC2, A K4, COX7C, MFN1, NDUFAB1, UQCRB, COX4I1
Mitochondrial translation	GO: 0032543	20	110	3.62E-21	MRPS34, MRPL28, MRPL32, MRPL27, C12orf65, MRPS25, MRPS28, MRPL58, MRPL57, TSFM, MRPS31, MRPS21, GADD45, GIP1, MRPL54, MRPL24, MRPL18, MRPS2, CHCHD1, MRPS26, MRPS33
Mitochondrial translational elongation	GO: 0070125	19	89	3.62E-21	MRPS34, MRPL28, MRPL32, MRPL27, MRPS25, MRPS28, MRPL58, MRPL57, GIP1, SFM, MRPS31, GADD45, MRPL54, MRPL24, MRPL18, MRPS2, CHCHD1, MRPS26, MRPS33, MRPS21
Mitochondrial translational termination	GO: 0070126	19	91	3.82E-21	MRPS34, MRPL28, MRPL32, MRPL27, MRPS2, C12orf65, MRPS25, MRPS21, MRPS28, MRPL58, MRPL57, MRPS31, GADD45, GIP1, MRPL54, MRPL24, MRPL18, CHCHD1, MRPS26, MRPS33
Oxidation reduction process	GO: 0055114	39	932	4.34E-21	UQCRC1, TXN2, IDH3G, NDUFS7, NDUFAF2, COX5B, PRDX5, NDUFB9, DLAT, NDUFB8, GPD2, RTN4IP1, CYC1, COX5A, COX8A, CHCHD5, NDUFA12, COX4I1, COX7A2, COQ6, PTGES2, NDUFA8, NDUFAF5, PDHA1, IDH3B, COX4I2, TXNRD2, NDUFA11, FDXR, COX7B, NDUFA13, COX7C, UQCRB, NDUFAB1, SLC25A14

**TABLE 2:** KEGG pathway enrichment for 112 mitochondrial genes.

Term	ID	Input gene count	Background gene count	False discovery rate	Matching proteins in the network (labels)
Oxidative phosphorylation	hsa00190	26	131	1.74E-29	UQCRC1, ATP5F1D, NDUFS7, COX5B, COX17, NDUFB10, NDUFS6, NDUFB11, NDUFB9, NDUFB8, CYC1, COX5A, COX8A, NDUFA12, COX7A2, COX4I2, NDUFA8, COX7C, ATP5MC1, ATP5MC2, NDUFA11, COX7B, NDUFA13, UQCRB, COX4I1, NDUFAB1
Thermogenesis	hsa04714	30	228	1.74E-29	UQCRC1, ATP5F1D, NDUFS7, COX5B, COX17, NDUFB10, NDUFS6, NDUFB11, NDUFB9, NDUFAF2, NDUFB8, CYC1, COX5A, COX8A, NDUFA12, NDUFAF4, COX7A2, NDUFA8, COX4I2, NDUFAF5, COX16, ATP5MC1, ATP5MC2, COX7B, NDUFA13, COX7C, NDUFAB1, NDUFA11, UQCRB, COX4I1
Parkinson's disease	hsa05012	26	142	3.76E-29	UQCRC1, ATP5F1D, PPIF, NDUFS7, COX5B, NDUFB10, NDUFS6, NDUFB11, NDUFB9, NDUFB8, CYC1, COX5A, COX8A, NDUFA12, COX7A2, NDUFA8, COX4I2, ATP5MC1, ATP5MC2, NDUFA11, COX7B, NDUFA13, COX7C, UQCRB, COX4I1, NDUFAB1
Alzheimer's disease	hsa05010	26	168	1.42E-27	UQCRC1, ATP5F1D, NDUFS7, COX5B, IDE, NDUFB10, CYC1, NDUFS6, NDUFB11, NDUFB9, NDUFB8, COX5A, COX8A, NDUFA12, COX7A2, NDUFA8, COX4I2, ATP5MC1, ATP5MC2, NDUFA11, COX7B, NDUFA13, COX7C, UQCRB, COX4I1, NDUFAB1
Huntington's disease	hsa05016	26	193	2.93E-26	UQCRC1, ATP5F1D, PPIF, NDUFS7, COX5B, NDUFB10, NDUFS6, NDUFB11, NDUFB9, NDUFB8, CYC1, COX5A, COX8A, NDUFA12, COX7A2, NDUFA8, COX4I2, ATP5MC1, ATP5MC2, NDUFA11, COX7B, NDUFA13, COX7C, UQCRB, COX4I1, NDUFAB1
Non-alcoholic fatty liver disease (NAFLD)	hsa04932	22	149	5.40E-23	UQCRC1, NDUFS7, COX5B, NDUFB10, NDUFS6, NDUFB11, NDUFB9, NDUFB8, CYC1, COX5A, COX8A, NDUFA12, COX7A2, NDUFA8, COX4I2, NDUFA11, COX7B, COX7C, NDUFA13, UQCRB, COX4I1, NDUFAB1
Metabolic pathways	hsa01100	36	1,250	2.07E-15	UQCRC1, ATP5F1D, IDH3G, NDUFS7, COX5B, COX17, LIAS, DGUOK, NDUFB10, NDUFS6, NDUFB11, NDUFB9, DLAT, NDUFB8, CYC1, COX5A, COX8A, NDUFA12, COQ6, PTGES2, NDUFA8, COX4I2, PDHA1, IDH3B, ATP5MC1, ATP5MC2, AK4, NDUFA11, CKMT1A, COX7B, NDUFA13, COX7C, UQCRB, MMAB, COX4I1, NDUFAB1
Cardiac muscle contraction	hsa04260	11	76	1.41E-11	UQCRC1, COX5B, CYC1, COX5A, COX8A, COX7A2, COX4I2, COX7B, COX7C, UQCRB, COX4I1
Retrograde endocannabinoid signaling	hsa04723	11	148	9.19E-09	NDUFS7, NDUFB10, NDUFS6, NDUFB11, NDUFB9, NDUFB8, NDUFA12, NDUFA8, NDUFAB1, NDUFA11, NDUFA13
Ribosome	hsa03010	7	130	6.30E-05	MRPL28, MRPL32, MRPL27, MRPL24, MRPL18, MRPS2, MRPS21
Citrate cycle (TCA cycle)	hsa00020	4	30	0.00017	IDH3G, DLAT, PDHA1, IDH3B
Pyruvate metabolism	hsa00620	3	39	0.0069	DLAT, PDHA1, HAGH
Carbon metabolism	hsa01200	4	116	0.0178	IDH3G, DLAT, PDHA, IDH3B
2-Oxocarboxylic acid metabolism	hsa01210	2	17	0.0178	IDH3G, IDH3B

## Discussion

Despite the great improvements in diagnosis and therapy, cancer remains one of the most threatening human survivors around the world. The more sensitive, specific, and efficient biomarkers are still insufficient, and the underlying mechanism in cancer development is elusive. Cancer cells exhibit an altered redox status and metabolism, which are associated with mitochondria as they are the major sites of ROS generation and energy metabolism [22]. Yes-associated protein (YAP) is a transcriptional coactivator that is involved in mitochondrial regulation, such as promoting mitochondrial biogenesis in endothelial cells [23], to up-regulate the mitochondrial iron exporter genes in yeast [24], to regulate the mitochondrial respiratory function in rectal and colon cancer cells [25,26]. Despite the intensive observation, the YAP1-mediated mitochondrial dysfunction in human cancers is still elusive. In the current study, we integrated the public databases to investigate the expression pattern and the prognostic performance of YAP1 across human cancers. Here, we reported that the higher YAP1 expression was strongly associated with a poorer prognosis in ACC, LGG, and PAAD. Additionally, further multiplatform revealed a YAP1-associated PPI network that was specific to mitochondria in the three types of tumors. Thus, our study provided evidence of the carcinogenesis of YAP1 in human cancers and new insights into its mitochondrial-regulatory network of YAP1.

In this study, we first evaluated the transcriptional levels of YAP1 and its prognostic values in various types of cancers by the multiplatform (Oncomine, GEPIA, and DriverDBV3). Based on the Oncomine database, we found that YAP was highly expressed in colorectal cancer, lymphoma, brain and CNS cancer, gastric and pancreatic cancer (Figure 1A). However, analysis of the GEPIA database showed YAP was highly expressed in lymphoma, glioblastoma multiforme, pancreatic adenocarcinoma, stomach adenocarcinoma, and thymoma. The discrepancies in levels of YAP1 expression in cancer from different databases may be attributable to the data collection approaches and underlying mechanisms pertinent to different biological properties. Nevertheless, these two databases consistently revealed that that YAP1 was highly expressed in lymphoma and pancreatic adenocarcinoma (PAAD). Intriguingly, all the three databases (GEPIA, UALCAN, and DriverDBv3) demonstrated that increased YAP1 expression was correlated with poor prognosis in adrenocortical carcinoma (ACC), brain lower-grade glioma (LGG), and pancreatic adenocarcinoma (PAAD), suggesting YAP1 might be a prognostic biomarker in ACC, LGG, and PAAD. In line with our result, the level of YAP1 determined by quantitative RT-PCR was increased 2.5-fold in pancreatic tumors in comparison to the normal pancreas [27]. To evaluate the prognostic value of YAP1 mRNA, Yang et al have recently extracted the expression profiles of 172 patients and corresponding clinical data from TCGA and performed univariate and multivariate regression analysis [28]. Consistent with our result from multiplatform analysis, they found the high level of YAP1 mRNA was an independent biomarker for poor prognosis in pancreas tumors. Accordingly, these previous observations, together with our study, suggested that the YAP1 could be a promising therapeutic strategy to treat pancreatic cancer. Also, YAP1 has been implicated as an oncogene in LGG. Brent et al used the publicly available REMBRANDT database and found high levels of YAP1 mRNA expression were associated with aggressive molecular subsets of glioblastoma. Although there were significantly worse outcomes in the high-YAP1 group concerning median survival, the trends toward differences in survival did not reveal statistical significance [29]. In our study, the multiplatform analysis showed the high level of YAP1 mRNA was correlated with poor prognosis in LGG. The disparity of prognostic value of YAP1 between Brent’s study and ours may attribute to the different sample sizes of the cases selected from the respective databases. Consistent with our result, Sang et al performed an immunohistochemistry assay in 141 cases of LGG and found YAP1 and pSTAT3-S727 co-expression might serve as a reliable prognostic biomarker and therapeutic target for glioma [30]. Taken together, these results indicated that YAP1 expression may serve as a potential diagnostic biomarker in these specific types of cancers.

In parallel, we identified the YAP1-coexpressed genes and screened the important modules and biological functions in the YAP1-associated tumors. Given the prognostic values in ACC, LGG, and PAAD, we screened the common YAP1-coexpressed genes in the three types of cancers, which were annotated by the Metascape and DAVID database, respectively. Our enrichment results revealed the YAP1 co-expression genes were significantly involved in citric acid (TCA) cycle and respiratory electron transport, PID FAK pathway, and signaling by receptor tyrosine kinases. These findings suggested that YAP1 played a crucial role in cancer metabolism (Figure 5). In accordance with our enrichment results, a previous study reported the YAP1-TEAD1 signaling induced mitochondrial biogenesis and increased oxygen consumption and glycolytic flux in endothelial cells, thereby promoting progression and cancer metastasis [23]. Besides, YAP was shown to act as a promoter of focal adhesion and tumor invasiveness *via* regulating FAK phosphorylation, in breast cancer and to induce FAK phosphorylation through a TEAD-dependent manner [31]. In addition to the FAK pathway, internal signals are capable of modulating the YAP1 pathway. One of the key initiators of internal signaling networks in cell proliferation and growth is the family of receptor tyrosine kinases (RTKs), which carries out the Hippo/YAP1 pathway by two main RTKs—the RTK/RAS/PI3K and the RTK-RAS-MAPK pathways [32]. On the other hand, several RTKs, including GFR, RET, and MERTK, have been shown to directly interact with and phosphorylate YAP/TAZ at multiple tyrosine residues independent of upstream Hippo signaling, thereby activating their functions in tumorigenesis [33]. Thus, the interactions between the Hippo/YAP1 and other signals (including RTKs) are complicated. Due to the complicated cellular functions of YAP1, there seems to be a multi-dimensional reciprocal regulatory mechanism between YAP1 and other signals.

Another important aspect of this study was that we constructed a YAP1-associated PPI network that is specific to mitochondria and identified hub genes in ACC, LGG, and PAAD. The network consisted of 112 mitochondria genes that created using reliable interactions from the open-source datasets (GO, IMPI, Mitocarta). These mitochondria genes have given enriched processes, functions, cell components, and KEGG pathways. “Oxidative phosphorylation (OXPHOS)” is the highest enriched molecular function, indicating the potential mechanism in YAP1-associated tumors. OXPHOS has been recently shown to be upregulated in certain cancers including pancreatic tumors [34]. Previous transcriptomic and metabolic analyses of PAAD stem-like cells revealed a strong reliance on OXPHOS and decreased glycolysis. Treatment with metformin or the complex V inhibitor oligomycin inhibited the growth of these stem-like cells *in vitro* and resulted in the growth delay of PDAC-215 and PDAC-A6L xenografts [35]. Consequently, the OXPHOS inhibitors could be used to target cancer cell metabolism in PAAD where OXPHOS is upregulated. Interestingly, recent observation revealed YAP1 was involved in the anti-cancer effect of OXPHOS inhibitors (metformin plus LW6) [36]. In the study, the authors demonstrated that metformin in combination with LW6 impaired pancreatic cancer cells and inhibited nuclear localization of YAP1 by phosphorylation of YAP1 at serine 127. Thus, OXPHOS regulates the expression and location of YAP1. On the other hand, YAP1 plays important role in maintaining redox balance in tumor cells. A previous study showed YAP1 was responsible for the metabolic switch from aerobic glycolysis to OXPHOS to drive up ROS levels in tumor cells [37]. Similarly, YAP1 was previously shown to promote the transcription of genes involved in mitochondrial fusion or fission [38]. In line with the result, our study showed the YAP1-coexpressed genes were enriched in mitochondrial gene expression (Table 1), indicating a strong correlation between YAP1 expression and mitochondrial homeostasis. Mitochondrial ribosomal proteins (MRPs) are essential components for the structural and functional integrity of the mitoribosome complex. In the study, several MRPs were associated with YAP1. Among these MRPs, MRPS37 (alias: CHCHD1) was identified as the seed gene in the 112 YAP1-associated genes (cluster1: Supplementary Table S4). Based on the GO enrichment, CHCHD1 was involved in mitochondrial gene expression, mitochondrial translation, mitochondrial translational elongation, and mitochondrial translational termination (Table 1). The result was in accordance with the previous observation that originally identified and characterized CHCHD1 as a member of the mammalian mitochondrial ribosome [39]. Given that the mitochondrial translational machinery and its components are vital for the expression of OXPHOS subunits, investigations of the associations between the translational component CHCHD1 and YAP1 have the utmost importance in understanding energy production by OXPHOS.

Here, we used several online databases to perform a comprehensive bioinformatics analysis of YAP1 expression and its potential mechanism in different types of tumors. The advantages of this method are its access to a massive sample population, lower cost, and large-scale genomic research and functional analysis capabilities. However, a few limitations inevitably existed in our study. First of all, due to the various collected sample sizes, the results of bioinformatics analysis varied across different databases. To eliminate the disparities between databases, we used the intersections of multiple databases. Take the prognostic value of YAP1 for example, we used three online databases (GEPIA, UALCAN, DriverDBv3), and we found that all three databases demonstrated YAP1 had prognostic potential in the ACC, LGG, and PAAD. Secondly, this study only displayed bioinformatics analysis findings based on different online databases. Therefore, further verification experiments including RT-PCR, Western Blot, as well as immunohistochemical experiments, are needed to verify the findings of the present study.

## Conclusion

YAP1 is a downstream effector of the Hippo pathway. Its activation and overexpression are associated with tumor development and progression. In the current study, we conducted a systemic analysis of the expression and the prognostic value of YAP1 in different types of cancer. Our results showed that the higher YAP1 expression was significantly associated with poorer survival in ACC, LGG, and PAAD. Given the comprehensive bioinformatics analysis, we constructed a YAP1 co-expressed gene network that is specific to mitochondria in ACC, LGG, and PAAD. Thus, our findings provided evidence of the carcinogenesis of YAP1 in human cancers and new insights into the mechanism underlying the function of YAP1 in mitochondrial dysfunction.

## Data Availability

The original contributions presented in the study are included in the article/Supplementary Material, further inquiries can be directed to the corresponding author.
