# Supplementary figures and images for: Gene Regulation Network of Prognostic Biomarker YAP1 in Human Cancers: An Integrated Bioinformatics Study
Source: Pathol Oncol Res. 2021 Jun 11;27:1609768. doi: 10.3389/pore.2021.1609768 (PMC8262238; doi:10.3389/pore.2021.1609768)

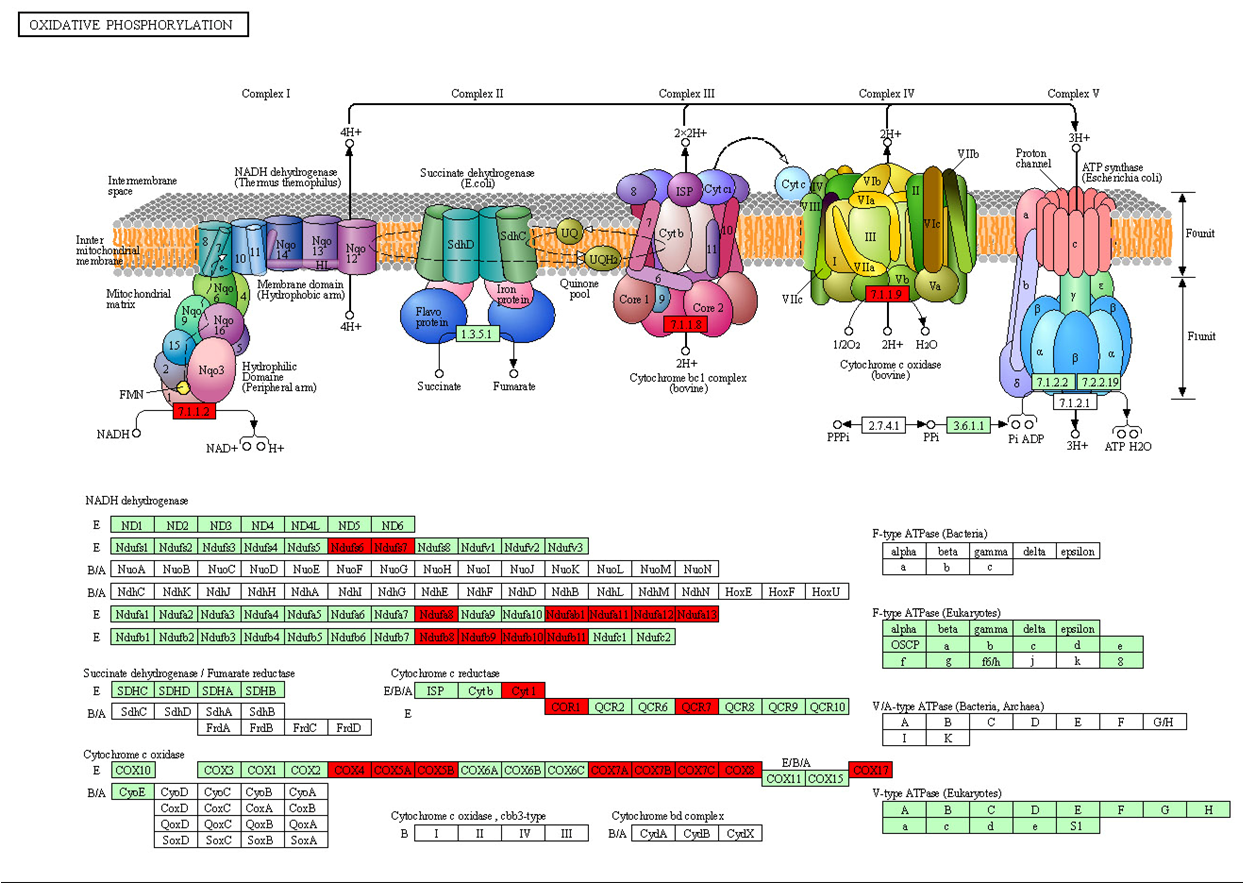

Supplement: Supplementary file 3 [file Image2.TIF]

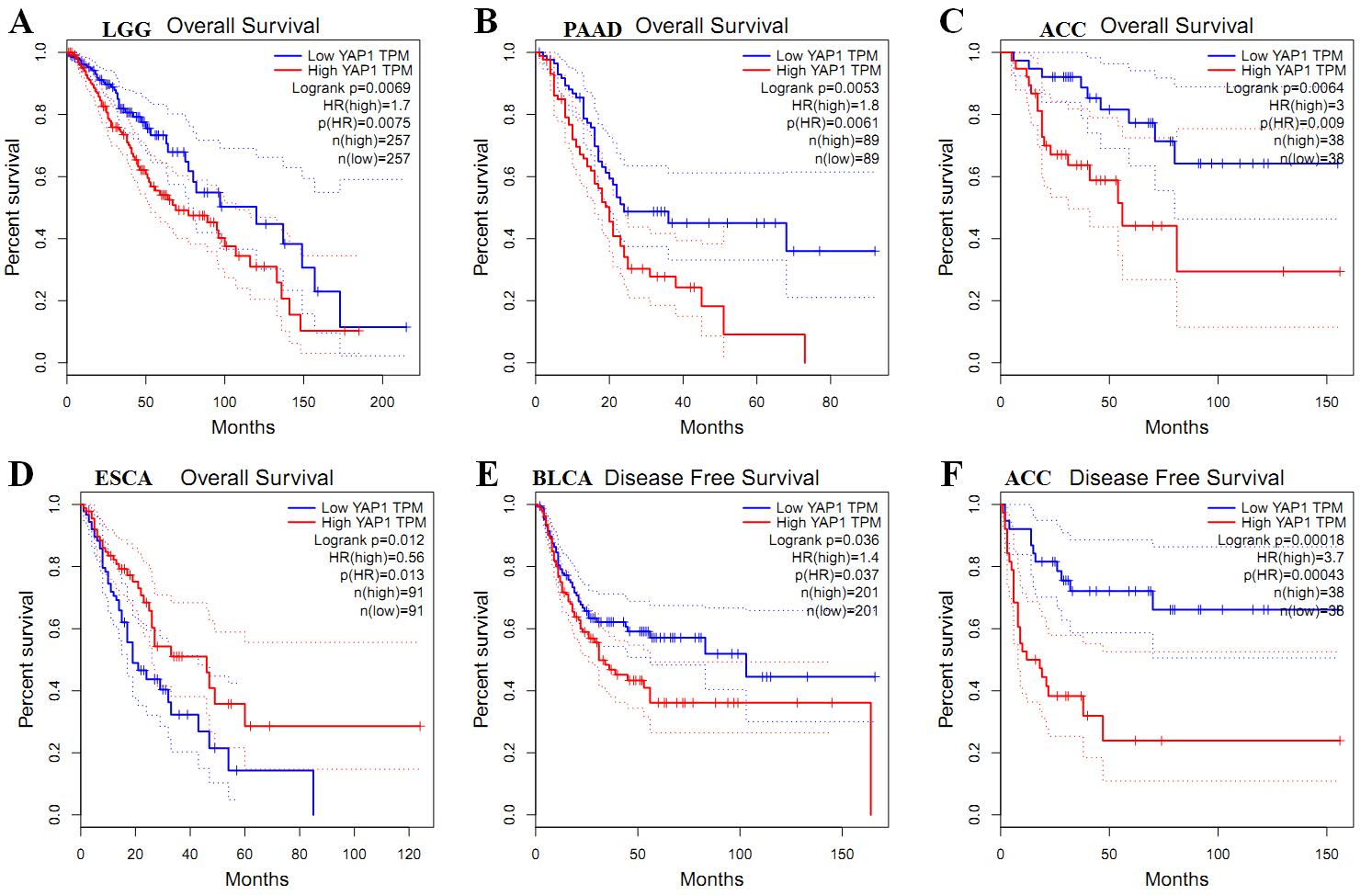

Supplement: Supplementary file 4 [file Image1.TIF]
